# Endocrine-related osteoporosis: bone quality, clinical phenotype, and targeted management

**DOI:** 10.3389/fendo.2026.1869556

**Published:** 2026-07-31

**Authors:** Ivan Raffaele Cincione, Roberto Marcantonio, Marcellino Monda, Rita Polito, Maria Casillo, Antonietta Messina, Antonietta Monda, Fiorenzo Moscatelli, Giovanni Messina, Annalisa Panico

**Affiliations:** 1 Department of Clinical and Experimental Medicine, University of Foggia, Foggia, Italy; 2 Prevention and Protection Area, University of Naples Federico II, Naples, Italy; 3 Unit of Dietetics and Sports Medicine, Department of Experimental Medicine, Section of Human Physiology, Università degli Studi della Campania “Luigi Vanvitelli”, Naples, Italy; 4 Department of Psychology and Health Sciences, Pegaso Telematic University, Naples, Italy; 5 Department of Precision Medicine, University of Campania “Luigi Vanvitelli”, Naples, Italy; 6 Department of Human Science and Quality of Life Promotion, San Raffaele Telematic University, Rome, Italy; 7 Department of Education and Sport Sciences, Pegaso Telematic University, Naples, Italy; 8 Loreto Crispi Endocrinology Unit, ASL Napoli 1 Centro, Naples, Italy

**Keywords:** bone quality, endocrine osteoporosis, fracture risk, secondary osteoporosis, trabecular bone score

## Abstract

Secondary osteoporosis is a major but frequently under-recognized contributor to skeletal fragility, particularly in men, premenopausal women, patients with fragility fractures at unexpectedly preserved bone mineral density, and individuals with rapid bone loss or inadequate response to standard therapy. Endocrine disorders are among the leading causes because they affect bone remodeling, mineral metabolism, sex steroid signaling, glucocorticoid pathways, insulin-like growth factor activity, and parathyroid hormone regulation. Management requires both correction of the underlying endocrine disorder and pharmacological treatment of skeletal fragility according to fracture risk. This review provides a clinically oriented and guideline-informed synthesis of endocrine-related secondary osteoporosis, with a focus on disease-specific skeletal phenotypes, diagnostic red flags, and targeted management strategies. Particular emphasis is placed on the concept of bone quality as a major determinant of fracture risk and on the limitations of conventional tools such as BMD and FRAX in this setting. We propose a practical clinical framework that integrates endocrine phenotype, vertebral imaging, trabecular bone score, and biochemical assessment into fracture-risk stratification and therapeutic decision-making. This approach supports a shift from a purely densitometric model toward a mechanism-based strategy, enabling improved identification of high- and very-high-risk patients and facilitating the appropriate use of anabolic and antiresorptive therapies.

## 1 Introduction

Osteoporosis is traditionally defined as a disorder characterized by low bone mass and microarchitectural deterioration, leading to increased fracture risk. Despite advances in fracture-risk assessment, a substantial proportion of fragility fractures still occur in individuals who do not meet densitometric criteria for osteoporosis, highlighting a critical gap in current diagnostic paradigms. Although primary postmenopausal and age-related osteoporosis remain the most common clinical entities, secondary osteoporosis represents a large and clinically important proportion of skeletal fragility. Secondary causes may be identified in up to 30% of postmenopausal women, more than 50% of premenopausal women, and 50-80% of men with osteoporosis (1). These estimates are particularly relevant because secondary osteoporosis often affects individuals not traditionally perceived as being at high skeletal risk, including younger adults, men, and those without classical risk factors. Endocrine diseases are among the most relevant causes of secondary osteoporosis. Bone is a target tissue for multiple hormonal systems, including parathyroid hormone, thyroid hormones, glucocorticoids, sex steroids, growth hormone, insulin-like growth factor 1, insulin, and mineralocorticoids. Alterations in these axes may impair bone remodeling, calcium-phosphate homeostasis, muscle function, fall risk, and skeletal microarchitecture (2, 3). These findings suggest that endocrine-related skeletal fragility cannot be fully explained by bone mass and instead requires integration of systemic hormonal effects on skeletal integrity. Although bone mineral density (BMD) remains essential for diagnosis and monitoring, it does not capture key determinants of bone strength, including microarchitecture, cortical porosity, collagen properties, and osteocyte function. Consequently, fragility fractures may occur at higher BMD values than expected, particularly in glucocorticoid-induced osteoporosis, type 2 diabetes mellitus, acromegaly, and primary aldosteronism (1, 3). This discordance between bone mass and fracture risk highlights a major limitation of current clinical practice and supports the need for complementary approaches capable of identifying disease-specific skeletal vulnerability.

**Figure 1:**
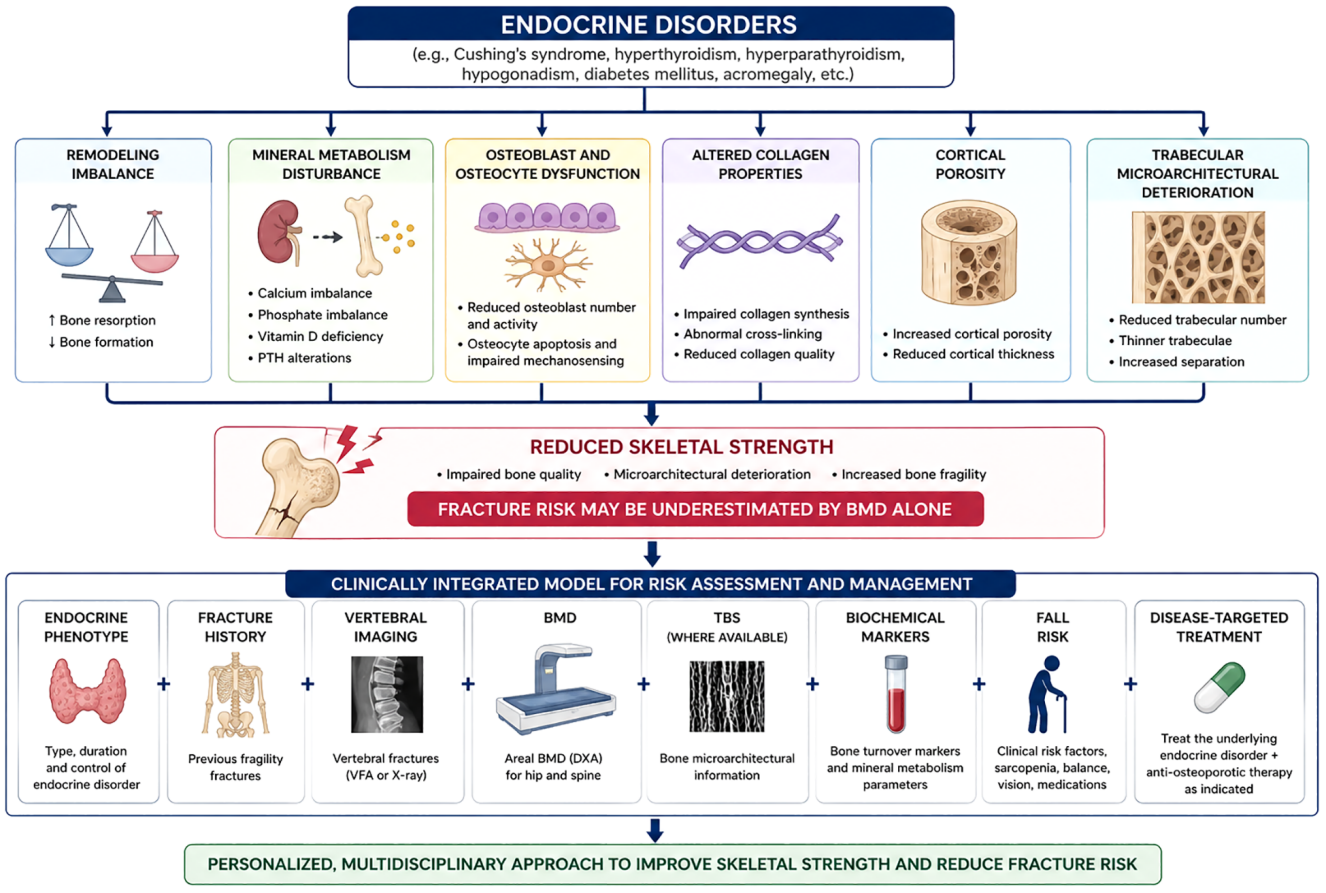
Pathophysiological framework linking endocrine disorders to skeletal fragility and integrated fracture risk assessment. The figure summarizes the main mechanisms through which endocrine disorders, including Cushing’s syndrome, hyperthyroidism, hyperparathyroidism, hypogonadism, diabetes mellitus, acromegaly, and other endocrine conditions, may compromise skeletal health. These disorders can affect bone through multiple and often overlapping pathways, including remodeling imbalance, characterized by increased bone resorption and/or reduced bone formation; disturbances in mineral metabolism, involving calcium and phosphate imbalance, vitamin D deficiency, and parathyroid hormone (PTH) alterations; osteoblast and osteocyte dysfunction, with reduced osteoblast number or activity and impaired osteocyte viability and mechanosensing; altered collagen properties, including impaired collagen synthesis, abnormal cross-linking, and reduced matrix quality; increased cortical porosity with reduced cortical thickness; and trabecular microarchitectural deterioration, characterized by reduced trabecular number, thinner trabeculae, and increased trabecular separation. The convergence of these mechanisms leads to reduced skeletal strength, impaired bone quality, microarchitectural deterioration, and increased bone fragility. Importantly, fracture risk may be underestimated when assessed by bone mineral density (BMD) alone, as BMD does not fully capture alterations in bone material properties, cortical and trabecular architecture, or endocrine-specific effects on bone quality. The lower panel illustrates a clinically integrated model for fracture risk assessment and management, combining endocrine phenotype, fracture history, vertebral imaging, areal BMD measured by dual-energy X-ray absorptiometry (DXA), trabecular bone score (TBS), biochemical markers of bone turnover and mineral metabolism, fall risk assessment, and disease-targeted treatment. VFA, vertebral fracture assessment; DXA, dual-energy X-ray absorptiometry; TBS, trabecular bone score; BMD, bone mineral density; PTH, parathyroid hormone.

This review provides a clinically oriented and guideline-informed synthesis of endocrine-related secondary osteoporosis, with emphasis on disease-specific skeletal phenotypes, diagnostic red flags, and targeted management strategies. The novelty of this review lies in the integration of two complementary perspectives: first, a disease-specific skeletal phenotype approach, which recognizes that each endocrine disorder produces a characteristic pattern of remodeling imbalance, mineral metabolism disturbance, microarchitectural impairment, or altered bone material properties; and second, a bone-quality-centered framework, which combines endocrine phenotype, vertebral imaging, BMD, TBS, biochemical assessment, fall risk, and advanced imaging tools into a multidimensional model of fracture-risk stratification. This approach aims to move beyond a purely densitometric interpretation of osteoporosis and to support a more individualized, mechanism-based strategy for diagnosis and management. The proposed conceptual framework is summarized in **Figure 1**.

## 2 From bone mineral density to bone quality in endocrine osteoporosis

Bone strength depends on both bone quantity and qualitative determinants of skeletal integrity. BMD reflects bone mineral content per projected area and is strongly associated with fracture risk at the population level. However, bone is a hierarchical three-dimensional structure whose resistance to fracture depends on geometry, cortical thickness, trabecular number and connectivity, mineralization, collagen cross-linking, osteocyte function, and microdamage repair (4).

Bone quality is not a single measurable parameter but rather a multidimensional construct. It includes microarchitecture, material properties, turnover, accumulated damage, mineral-to-matrix ratio, and spatial distribution of mineralization. These dimensions may be altered even when BMD is normal or only mildly reduced. This concept explains the frequent discordance between bone mass and skeletal fragility observed in endocrine-related secondary osteoporosis. Several technologies can provide indirect or direct information on bone quality. Trabecular bone score (TBS) is a DXA-derived texture index that reflects gray-level variation in lumbar spine images and provides indirect information on trabecular microarchitecture. TBS predicts fracture risk independently of BMD and may improve risk estimation when combined with BMD and clinical risk factors (5–7). Quantitative computed tomography, peripheral quantitative computed tomography, high-resolution peripheral quantitative computed tomography, and magnetic resonance imaging can further characterize volumetric density, cortical geometry, and trabecular structure. Among these techniques, TBS is currently the most accessible tool in routine clinical practice and provides a pragmatic approach to incorporating microarchitectural information into fracture-risk assessment. More recently, radiofrequency echographic multi-spectrometry (REMS) has been proposed as a radiation-free ultrasound-based technique for assessing bone status, with emerging evidence suggesting its potential role in fracture-risk evaluation beyond conventional densitometric measures (8). However, the role of REMS in endocrine-related secondary osteoporosis remains incompletely defined. REMS is a radiation-free, ultrasound-based technology capable of evaluating the lumbar spine and proximal femur and may represent an attractive alternative or complementary tool in situations where repeated assessments are required or DXA accessibility is limited. Preliminary studies suggest that REMS may improve fracture-risk discrimination beyond conventional BMD measurements; however, endocrine-specific validation studies remain limited, and further research is needed before its routine incorporation into endocrine osteoporosis algorithms.

Additional imaging approaches may further enhance skeletal phenotyping without substantially increasing radiation exposure. Three-dimensional DXA (3D-DXA) is an emerging technique that reconstructs volumetric information from conventional DXA acquisitions, providing estimates of cortical thickness, trabecular density, and proximal femoral geometry. These parameters may offer clinically relevant insights into skeletal strength beyond areal BMD and may be particularly useful in endocrine disorders characterized by discordance between bone density and fracture risk. Lateral spine imaging and vertebral morphometry, including vertebral fracture assessment (VFA), also deserve particular consideration in endocrine osteoporosis. These techniques allow identification of prevalent vertebral fractures that frequently remain clinically silent, especially in glucocorticoid-induced osteoporosis, acromegaly, primary hyperparathyroidism, and diabetes mellitus. Because vertebral fractures are among the strongest predictors of future fracture risk, their systematic evaluation may substantially improve risk stratification even in patients with osteopenic or apparently normal BMD.

In endocrine osteoporosis, bone-quality assessment is particularly relevant because several endocrine disorders preferentially impair microarchitecture or material properties rather than bone mass. Trabecular perforation, cortical thinning, and cortical porosity may increase even when BMD is preserved. This mechanism is particularly relevant in high-turnover states, where accelerated remodeling leads to rapid microarchitectural deterioration. Consequently, a clinically comprehensive assessment model should combine disease context, fracture history, vertebral imaging, BMD, TBS where available, biochemical phenotype, and fall risk, moving beyond a purely densitometric approach.

Risk stratification in endocrine-related osteoporosis should integrate densitometric parameters with disease-specific and qualitative determinants of skeletal strength. A prior fragility fracture, especially a recent vertebral or hip fracture, is one of the strongest indicators of imminent fracture risk. Vertebral fractures are frequently asymptomatic and should be actively sought in high-risk endocrine settings. In this context, recent fractures identify patients at imminent risk who may benefit from early and intensive therapeutic intervention.

FRAX can support fracture-risk estimation but has limitations in secondary osteoporosis. It does not fully capture glucocorticoid dose and duration, diabetes severity, disease activity, microvascular complications, acromegaly activity, or aldosteronism-related calcium metabolism. Adjustments have been proposed for glucocorticoid dose and diabetes, but clinical judgment remains essential. This limitation reinforces the need for clinical judgment and disease-specific risk assessment beyond standardized tools.

TBS may improve risk classification when densitometric findings and clinical phenotype are discordant. This is especially important when patients have fragility fractures with osteopenic or normal BMD, or when endocrine disease is known to impair microarchitecture. Its use is particularly valuable in identifying patients whose fracture risk is underestimated by conventional densitometric evaluation. The 2024 UK/NOGG guideline emphasizes multidimensional risk assessment and intervention thresholds rather than reliance on BMD alone (32).

Patients should be considered at very high skeletal fragility when they have recent major osteoporotic fracture, multiple vertebral fractures, very low BMD, high-dose or prolonged glucocorticoid exposure, fracture during therapy, or severe endocrine disease with documented skeletal involvement. In such cases, anabolic-first or sequential anabolic-antiresorptive strategies may be appropriate. This stratification framework supports a personalized therapeutic approach aligned with the underlying pathophysiological mechanisms.

## 3 Shared endocrine mechanisms of skeletal fragility

Understanding these qualitative determinants of bone strength provides the basis for interpreting the diverse pathophysiological mechanisms through which endocrine disorders impair skeletal integrity. Endocrine disorders impair skeletal integrity through heterogeneous but converging pathophysiological mechanisms that extend beyond alterations in bone mass. Excess parathyroid hormone, thyroid hormone excess, glucocorticoid exposure, and inflammatory-metabolic activation may increase bone resorption through receptor activator of nuclear factor kappa-B ligand and osteoprotegerin imbalance. When resorption is not matched by adequate formation, trabecular perforation, cortical thinning, and cortical porosity increase.

A second pathway is suppression of bone formation. Glucocorticoids, hypogonadism, diabetes, and growth hormone deficiency may impair osteoblast differentiation, reduce osteoblast lifespan, and suppress matrix synthesis. Glucocorticoids are particularly important because they inhibit Wnt/β-catenin signaling, promote osteoblast and osteocyte apoptosis, and shift mesenchymal precursors toward adipogenesis (9, 10). Impaired bone formation represents a key determinant of skeletal fragility in several endocrine disorders and provides a rationale for considering anabolic therapeutic strategies in selected high-risk patients.

A third pathway is alteration of bone material properties. In diabetes mellitus, chronic hyperglycemia promotes accumulation of advanced glycation end-products in collagen, increasing stiffness but reducing toughness and resistance to microdamage. This may explain why patients with type 2 diabetes can have normal or increased BMD but elevated fracture risk. These alterations are not captured by conventional densitometric techniques, further contributing to the underestimation of fracture risk in these patients.

A fourth pathway is disruption of mineral metabolism. Primary hyperparathyroidism, secondary hyperparathyroidism associated with chronic kidney disease, vitamin D deficiency, hypoparathyroidism, and primary aldosteronism alter calcium balance, phosphate homeostasis, urinary calcium excretion, fibroblast growth factor 23 signaling, and parathyroid hormone activity. In CKD-MBD, phosphate retention, reduced calcitriol synthesis, and persistent PTH elevation may profoundly affect both cortical and trabecular bone compartments, contributing to skeletal fragility through mechanisms that extend beyond bone mass loss. In primary aldosteronism, hypercalciuria and secondary hyperparathyroidism have been proposed as central skeletal mechanisms (11).

**Figure 2:**
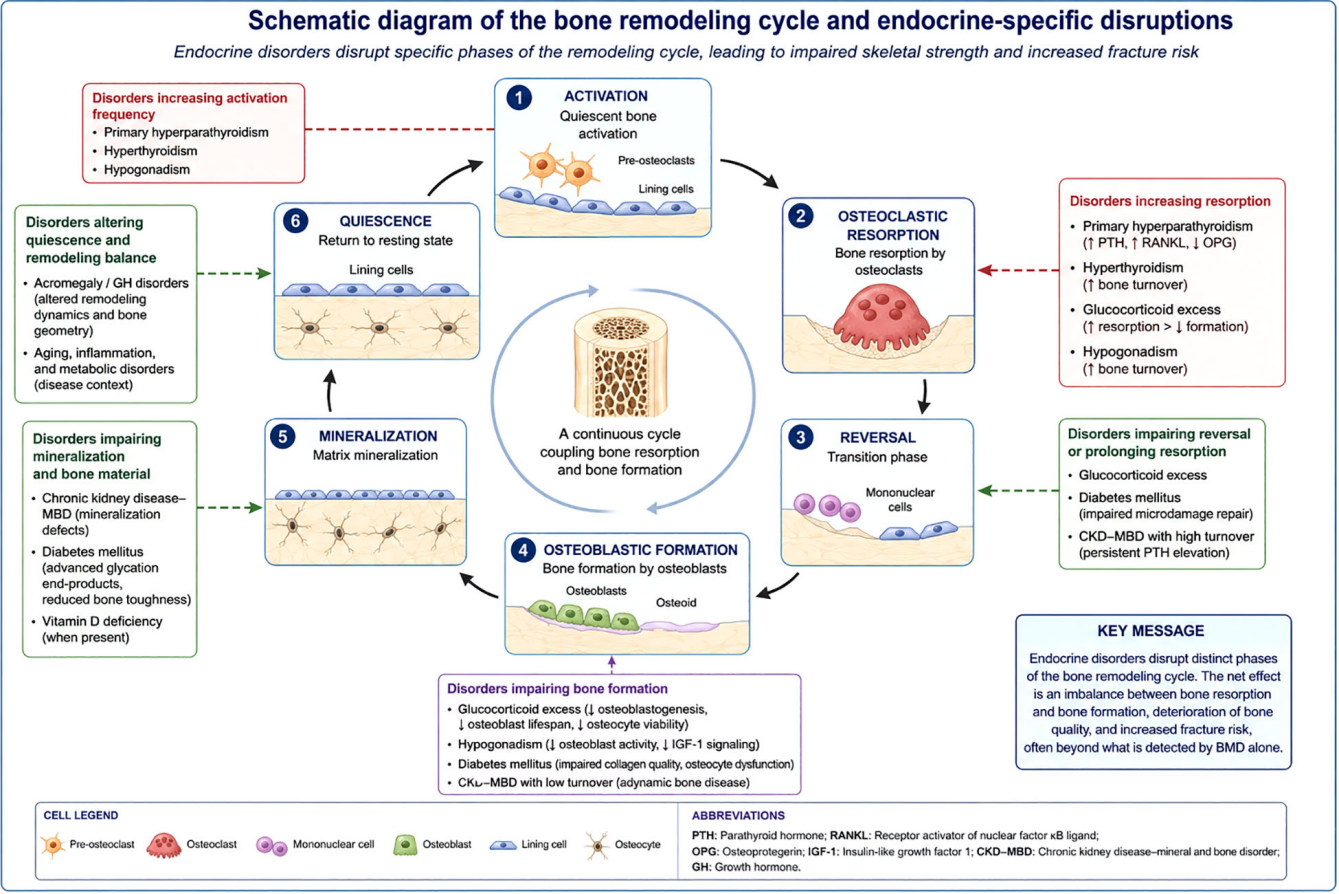
Schematic representation of the bone remodeling cycle and endocrine-specific disruptions contributing to skeletal fragility. Bone remodeling is a continuous and tightly regulated process consisting of six sequential phases: activation, osteoclastic resorption, reversal, osteoblastic formation, mineralization, and quiescence. Different endocrine disorders preferentially affect specific stages of this cycle, leading to alterations in bone quality, microarchitecture, and fracture susceptibility. Primary hyperparathyroidism and hyperthyroidism predominantly increase remodeling activation and osteoclastic resorption, resulting in accelerated bone turnover and structural deterioration. Glucocorticoid excess suppresses osteoblast differentiation and activity, promotes osteocyte apoptosis, and impairs bone formation. Hypogonadism contributes to both increased bone resorption and reduced bone formation through sex steroid deficiency. Diabetes mellitus affects bone material properties through advanced glycation end-product accumulation, impaired osteocyte function, and defective microdamage repair. Chronic kidney disease–mineral and bone disorder (CKD-MBD) may disrupt mineralization and produce either high-turnover or low-turnover skeletal phenotypes depending on disease stage and parathyroid hormone activity. Growth hormone disorders and acromegaly alter remodeling dynamics, skeletal geometry, and vertebral strength despite frequently preserved bone mineral density (BMD). The cumulative effect of these endocrine disturbances is an imbalance between bone resorption and bone formation, leading to deterioration of bone quality and increased fracture risk that may not be adequately captured by conventional densitometric assessment alone. PTH, parathyroid hormone; RANKL, receptor activator of nuclear factor kappa-B ligand; OPG, osteoprotegerin; IGF-1, insulin-like growth factor 1; CKD-MBD, chronic kidney disease–mineral and bone disorder; GH, growth hormone; BMD, bone mineral density.

Finally, endocrine disorders may increase fracture risk through extra-skeletal pathways, including sarcopenia, myopathy, neuropathy, visual impairment, hypoglycemia, postural instability, and falls. Fracture prevention in endocrine-related osteoporosis therefore requires a multidimensional approach integrating skeletal assessment with endocrine disease activity, systemic risk factors, and fall risk. **Figure 2** provides a schematic representation of the bone remodeling cycle and highlights the endocrine-specific disruptions that may affect each phase. Primary hyperparathyroidism and hyperthyroidism mainly increase remodeling activation and osteoclastic resorption, whereas glucocorticoid excess and hypogonadism impair osteoblast differentiation, matrix synthesis, and bone formation. Diabetes mellitus predominantly affects bone material properties, collagen quality, osteocyte function, and microdamage repair, while chronic kidney disease–mineral and bone disorder may alter mineralization and produce either high- or low-turnover skeletal phenotypes. Growth hormone disorders and acromegaly may further disrupt remodeling balance, bone geometry, and vertebral strength. This mechanism-based representation reinforces the concept that endocrine-related osteoporosis should be interpreted as a disorder of remodeling balance and bone quality rather than as a condition defined exclusively by BMD.

## 4 Disease-specific endocrine phenotypes

### 4.1 Glucocorticoid excess

Glucocorticoid-induced osteoporosis is the most common form of secondary osteoporosis. It may result from exogenous glucocorticoid therapy or endogenous hypercortisolism. Fracture risk increases rapidly after glucocorticoid initiation, often before major loss of bone mass is detectable, and vertebral fractures are particularly frequent (9–11).

The skeletal effects of glucocorticoids are biphasic. Early exposure increases bone resorption, whereas chronic exposure suppresses bone formation. Mechanisms include increased osteoclastogenesis through receptor activator of nuclear factor kappa-B ligand and macrophage colony-stimulating factor, reduced osteoprotegerin, inhibition of osteoblastogenesis, increased osteoblast and osteocyte apoptosis, suppression of insulin-like growth factor 1, inhibition of Wnt signaling, reduced intestinal calcium absorption, increased urinary calcium loss, and glucocorticoid-induced myopathy (9, 10). The clinical phenotype is characterized by rapid bone loss, vertebral predominance, asymptomatic vertebral fractures, and fractures occurring at higher densitometric values than in primary osteoporosis. Therefore, postmenopausal osteoporosis thresholds should not be applied mechanically to glucocorticoid-exposed patients. This phenotype is characterized by rapid bone loss, vertebral predominance, asymptomatic vertebral fractures, and fractures occurring at higher densitometric values than in primary osteoporosis (12, 13). Therefore, glucocorticoid-exposed patients require early skeletal assessment, including vertebral fracture evaluation, and should not be evaluated by applying postmenopausal osteoporosis thresholds mechanically.

### 4.2 Thyroid disorders

Overt hyperthyroidism produces a high-turnover skeletal state. Thyroid hormone excess shortens the remodeling cycle and increases the frequency of remodeling activation, with bone resorption exceeding bone formation. Cortical bone is particularly vulnerable, contributing to hip and non-vertebral fracture susceptibility.

Subclinical hyperthyroidism may also increase the risk of fragility fractures, especially in older adults and in patients with persistently suppressed thyrotropin. This is clinically relevant in endogenous thyroid autonomy and in iatrogenic thyrotoxicosis caused by excessive levothyroxine replacement or suppressive therapy (14, 15).

The skeletal phenotype includes accelerated bone turnover, cortical bone loss, increased hip fracture risk, and worsening of postmenopausal bone loss. Restoration of euthyroidism is the primary skeletal intervention.

### 4.3 Hypogonadism

Sex steroid deficiency is a major determinant of bone loss in both women and men. Estrogen deficiency increases osteoclast survival and bone resorption, whereas androgen deficiency contributes to reduced bone formation, reduced periosteal apposition, sarcopenia, and impaired mechanical loading. In men, estrogen derived from aromatization of testosterone is a key regulator of skeletal remodeling.

Hypogonadism should be actively considered in men with osteoporosis, low-trauma fractures, reduced libido, infertility, pituitary disease, chronic opioid exposure, androgen-deprivation therapy, or unexplained low BMD. In women, premature ovarian insufficiency, hypothalamic amenorrhea, eating disorders, early menopause, and anti-estrogen therapies are major risk contexts.

The 2024 evidence-based guideline for osteoporosis in men emphasizes systematic assessment of secondary causes, appropriate interpretation of densitometry, and treatment according to absolute fracture risk (16).

### 4.4 Primary hyperparathyroidism

Primary hyperparathyroidism is characterized by autonomous parathyroid hormone secretion, hypercalcemia or normocalcemic variants, and increased skeletal remodeling. Traditionally, primary hyperparathyroidism has been associated with preferential cortical bone loss, particularly at the distal one-third radius. However, modern imaging has shown that both cortical and trabecular compartments may be affected (17–19). The typical skeletal phenotype includes cortical thinning, reduced bone mass at forearm and hip, possible relative preservation of lumbar spine areal BMD, and enhanced skeletal fragility. TBS and high-resolution peripheral quantitative computed tomography have demonstrated microarchitectural deterioration even in patients with apparently mild disease (18, 19). The Fifth International Workshop guidelines define surgical criteria including symptomatic disease, fragility fracture, BMD T-score ≤ -2.5 at relevant sites, renal involvement, age below 50 years, and serum calcium above guideline thresholds (17). Parathyroidectomy may improve BMD and reduce skeletal risk, but residual fracture risk should be reassessed after biochemical cure. These findings support the concept that skeletal involvement in primary hyperparathyroidism extends beyond cortical bone loss and may not be fully captured by conventional densitometric assessment.

### 4.5 Secondary hyperparathyroidism and chronic kidney disease–mineral and bone disorder

Secondary hyperparathyroidism associated with chronic kidney disease (CKD) is a major and often under-recognized contributor to skeletal fragility. Progressive renal dysfunction leads to phosphate retention, impaired synthesis of active vitamin D, hypocalcemia, fibroblast growth factor 23 (FGF23) dysregulation, and compensatory increases in parathyroid hormone (PTH) secretion. These alterations constitute the pathophysiological basis of chronic kidney disease–mineral and bone disorder (CKD-MBD), a systemic condition affecting bone turnover, mineralization, bone volume, and extraskeletal calcification (20).

The skeletal phenotype of CKD-MBD is heterogeneous and may include high-turnover bone disease driven by persistent secondary hyperparathyroidism, low-turnover or adynamic bone disease, defective mineralization, cortical thinning, trabecular deterioration, and increased fracture susceptibility (21). Fracture risk is substantially increased across all stages of CKD and cannot be adequately explained by BMD alone. Indeed, alterations in bone quality, microarchitecture, and material properties frequently contribute to skeletal fragility despite apparently preserved densitometric values.

Assessment should include serum calcium, phosphate, alkaline phosphatase, 25-hydroxyvitamin D, PTH, and renal function parameters. Interpretation of bone turnover markers and imaging findings should always consider CKD stage, as the relationship between biochemical abnormalities and skeletal pathology may differ substantially from that observed in primary osteoporosis (22). Close collaboration between endocrinologists, nephrologists, and bone specialists is often required in advanced disease.

### 4.6 Diabetes mellitus

Diabetes mellitus is a paradigmatic example of dissociation between BMD and fracture risk. Type 1 diabetes is generally associated with lower BMD and increased fracture risk, partly because insulin deficiency and altered insulin-like growth factor signaling impair skeletal acquisition and remodeling. Type 2 diabetes often presents with normal or increased DXA-derived parameters but increased fracture susceptibility, particularly at the hip and vertebral sites (23–25). Several mechanisms explain diabetic bone fragility. Hyperglycemia induces advanced glycation end-products in collagen, reducing bone toughness. Bone turnover is often low, impairing microdamage repair. Microvascular disease, chronic inflammation, oxidative stress, renal dysfunction, and altered osteocyte function may further compromise skeletal integrity. In addition, retinopathy, neuropathy, hypoglycemia, sarcopenia, and impaired balance increase fall risk. FRAX may underestimate fracture risk in type 2 diabetes because diabetes is not fully represented as an independent disease-specific variable. TBS may improve assessment by capturing part of the microarchitectural impairment. Updated evidence suggests that type 2 diabetes is associated with lower TBS despite preserved or higher BMD, supporting the concept that diabetic bone disease is primarily driven by microarchitectural impairment rather than representing a simple low-BMD condition (26). Clinical assessment should integrate diabetes duration, glycemic control, microvascular complications, medications associated with falls or skeletal effects, vertebral fracture assessment, BMD, and TBS where available. Overall, diabetes-related skeletal fragility requires integrated assessment of metabolic control, microvascular complication, fall risk, vertebral imaging, and microarchitectural indices where available.

### 4.7 Growth hormone disorders and acromegaly

Growth hormone and insulin-like growth factor 1 regulate bone growth, bone turnover, muscle mass, and skeletal remodeling. Growth hormone deficiency may reduce bone formation and impair skeletal strength. Acromegaly, in contrast, is associated with increased bone turnover, altered bone geometry, and increased vertebral susceptibility despite normal or increased BMD.

Vertebral fractures may occur even when DXA does not show osteoporosis. Contributing factors include disease duration, hypogonadism, impaired glucose metabolism, altered trabecular microarchitecture, and persistent growth hormone/insulin-like growth factor 1 excess. Recent evidence suggests that vertebral fracture risk in acromegaly remains clinically relevant regardless of BMD, and that TBS interpretation may be affected by soft tissue thickness and glucose metabolism (27).

Vertebral fracture assessment should therefore be considered in acromegaly, especially in patients with active disease, hypogonadism, impaired glucose metabolism, back pain, height loss, or long disease duration.

### 4.8 Primary aldosteronism

Primary aldosteronism is increasingly recognized as a secondary cause of bone fragility. Traditionally approached as a cardiovascular and renal disorder, it may also affect calcium metabolism, parathyroid hormone secretion, and bone microarchitecture. A systematic review and meta-analysis showed that patients with primary aldosteronism have higher parathyroid hormone levels and increased urinary calcium excretion compared with essential hypertension controls. The same synthesis reported an association with increased risk of fragility fractures and suggested that skeletal alterations may improve after adrenalectomy or mineralocorticoid receptor antagonist therapy (28).

The mechanisms linking aldosterone excess to skeletal fragility include renal calcium loss, secondary hyperparathyroidism, oxidative stress, inflammation, and possible direct mineralocorticoid receptor-mediated effects on bone cells. Importantly, DXA may underestimate skeletal involvement because primary aldosteronism may preferentially impair bone microarchitecture rather than bone mass. Lower TBS has been reported in patients with primary aldosteronism despite no major BMD differences, supporting a phenotype characterized by microarchitectural impairment (29). A population-based cohort study also found increased fracture risk in primary aldosteronism (30), while clinical data support considering primary aldosteronism as a potentially reversible cause of secondary osteoporosis (31).

Primary aldosteronism should be suspected in patients with resistant hypertension, hypokalemia, adrenal incidentaloma, early-onset hypertension, or hypertension associated with unexplained skeletal fragility, hypercalciuria, or elevated parathyroid hormone. **Table 1** summarizes the main endocrine skeletal phenotypes discussed in this review. This condition represents a potentially reversible model of endocrine-related skeletal fragility, in which early recognition may significantly impact both cardiovascular and skeletal outcomes.

**Table 1:** Endocrine skeletal phenotypes.

Disorder	Typical BMD pattern	Dominant bone-quality issue	Main mechanism	Reversibility
Glucocorticoid excess	Normal, osteopenic, or osteoporotic	Trabecular deterioration, osteocyte apoptosis	Low bone formation, early resorption increase, Wnt inhibition	Partial; depends on dose reduction and treatment
Hyperthyroidism	Reduced, especially cortical sites	High-turnover cortical loss	Accelerated remodeling cycle	Often improves with euthyroidism
Hypogonadism	Reduced spine/hip BMD	Increased remodeling, sarcopenic component	Estrogen/androgen deficiency	Partial with hormonal correction and skeletal therapy
Secondary hyperparathyroidism/CKD-MBD	Variable; may be normal, reduced, or discordant with fracture risk	Cortical deterioration, mineralization defects, high- or low-turnover bone disease	Phosphate retention, vitamin D deficiency, FGF23 dysregulation, persistent PTH elevation	Partially reversible through correction of CKD-MBD abnormalities and optimized disease management
Primary hyperparathyroidism	Cortical BMD loss; distal radius often affected	Cortical thinning, trabecular involvement possible	PTH-mediated high turnover	Often improves after parathyroidectomy
Type 2 diabetes	Normal or increased BMD	Collagen glycation, low turnover, microvascular damage	AGEs, low remodeling, falls	Limited; risk reduction through integrated management
Acromegaly	Normal or increased BMD possible	Vertebral fragility despite preserved BMD	GH/IGF-1 excess, hypogonadism, glucose impairment	Partial with biochemical control
Primary aldosteronism	Often normal or mildly reduced BMD	Reduced TBS, microarchitectural impairment	Hypercalciuria, secondary hyperparathyroidism, aldosterone effects	Potentially reversible with adrenalectomy/MR antagonists

This table summarizes the main endocrine disorders associated with secondary osteoporosis, highlighting their characteristic skeletal phenotypes. For each condition, the table reports the typical bone mineral density (BMD) pattern, the dominant alteration in bone quality, the underlying pathophysiological mechanism, and the potential reversibility of skeletal involvement following treatment of the endocrine disorder. This synthesis underscores the heterogeneity of endocrine-related skeletal fragility and emphasizes the frequent dissociation between BMD and fracture risk. By integrating densitometric findings with qualitative and mechanistic determinants of bone strength, the table supports a multidimensional, mechanism-based approach to fracture risk assessment and personalized management.

## 5 Diagnostic strategy for suspected endocrine secondary osteoporosis

The diagnostic approach to endocrine-related secondary osteoporosis should begin with a high index of clinical suspicion. Endocrine-related secondary osteoporosis should be considered in men with osteoporosis, premenopausal women with low BMD or fragility fractures, patients younger than expected for primary osteoporosis, individuals with rapid bone loss, patients with multiple fractures, and those with fracture history discordant with densitometric parameters. Key clinical red flags are summarized in **Table 2**.

**Table 2:** Clinical red flags suggesting endocrine-related secondary osteoporosis.

Clinical feature	Diagnostic implication	Suggested action
Osteoporosis in men	Secondary cause more likely than in postmenopausal women	Screen for hypogonadism, hyperparathyroidism, thyroid disease, diabetes, glucocorticoid exposure, renal and liver disease
Fragility fracture in premenopausal women or adults <50 years	Primary osteoporosis less likely	Perform extended endocrine and metabolic evaluation
Z-score ≤ -2.0	Low bone mass for age	Investigate secondary causes
Vertebral fracture with osteopenic or normal BMD	BMD-fracture mismatch	Add vertebral imaging, TBS if available, endocrine work-up
Rapid bone loss	High-turnover or drug-induced condition	Assess thyroid status, PTH, glucocorticoids, malabsorption, inflammatory disease
Long-term glucocorticoid exposure	High early vertebral fracture risk	Early risk stratification and treatment according to GIOP guidelines
Type 2 diabetes with fracture despite preserved BMD	Bone-quality impairment likely	Consider TBS, vertebral imaging, fall-risk evaluation
Resistant hypertension or hypokalemia with skeletal fragility	Possible primary aldosteronism	Measure aldosterone-renin ratio
Acromegaly or pituitary disease	Vertebral fracture risk may be underestimated by BMD	Assess IGF-1, gonadal axis, vertebral fractures
Poor response to anti-osteoporotic therapy	Unrecognized secondary driver possible	Reassess endocrine and metabolic causes

This table summarizes key clinical features that should raise suspicion for endocrine-related secondary osteoporosis in routine practice. These “red flags” are particularly relevant in patients whose fracture risk appears disproportionate to bone mineral density (BMD) or in clinical contexts atypical for primary osteoporosis. For each feature, the table outlines the corresponding diagnostic implication and the recommended clinical action, supporting a structured and phenotype-driven diagnostic approach. The identification of these warning signs is essential to prompt timely endocrine and metabolic evaluation, enabling the recognition of potentially reversible causes of skeletal fragility and guiding appropriate diagnostic and therapeutic strategies.

**Figure 3:**
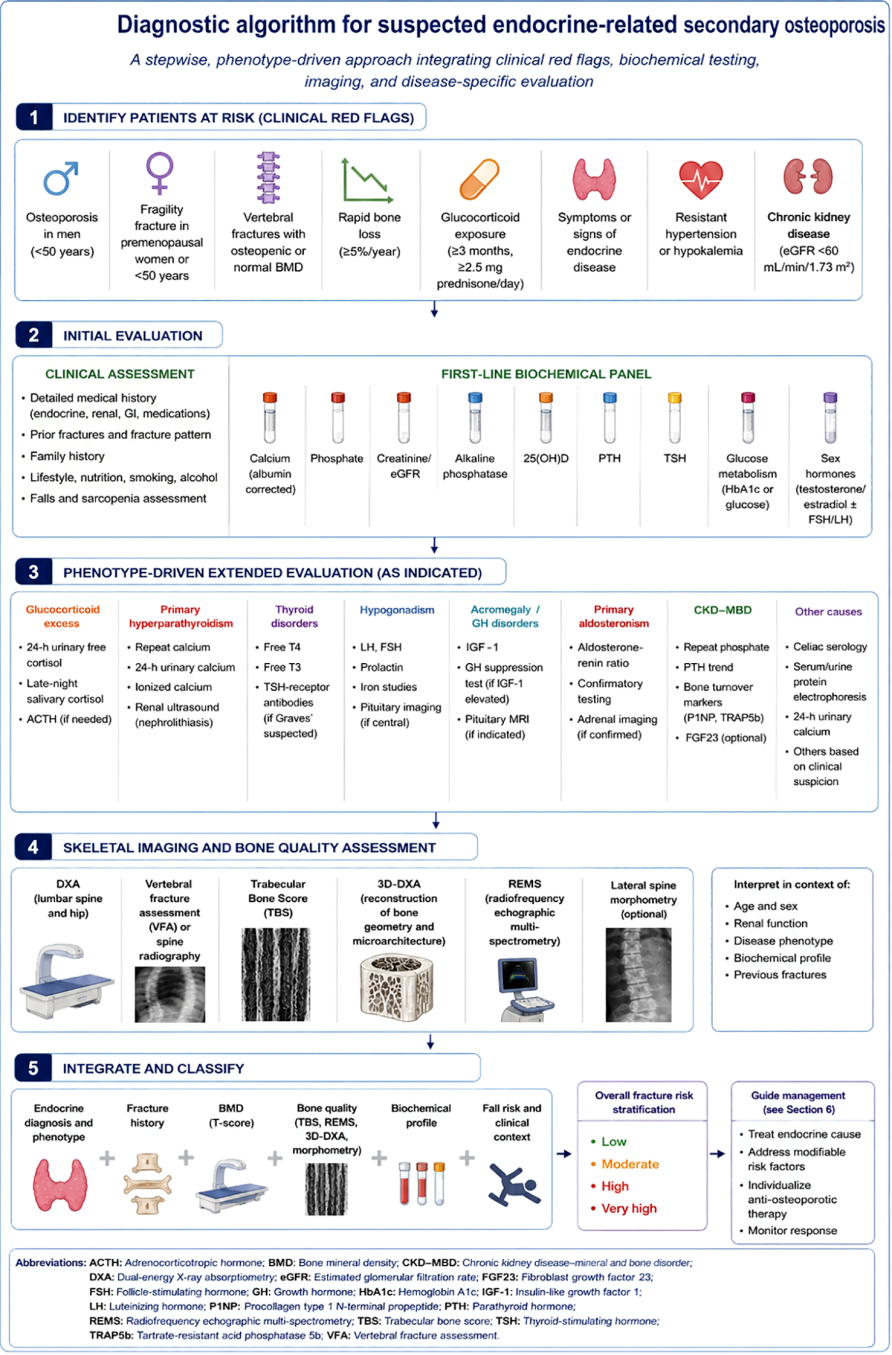
Diagnostic algorithm for suspected endocrine-related secondary osteoporosis. The algorithm illustrates a stepwise, phenotype-driven approach for the evaluation of patients with suspected endocrine-related skeletal fragility. The process begins with the identification of clinical red flags, including osteoporosis in men, fragility fractures in premenopausal women or younger adults, vertebral fractures disproportionate to BMD, rapid bone loss, glucocorticoid exposure, symptoms suggestive of endocrine disease, resistant hypertension, or chronic kidney disease. Initial assessment includes clinical evaluation and a first-line biochemical panel comprising calcium, phosphate, renal function, alkaline phosphatase, 25-hydroxyvitamin D, parathyroid hormone, thyroid-stimulating hormone, glucose metabolism assessment, and sex hormone evaluation when appropriate. Subsequent investigations are guided by the suspected endocrine phenotype and may include cortisol testing, urinary calcium, aldosterone-renin ratio, insulin-like growth factor 1, celiac screening, or myeloma evaluation. Skeletal imaging integrates DXA, vertebral fracture assessment, trabecular bone score, and advanced techniques such as REMS, 3D-DXA, and lateral spine morphometry when clinically indicated. The final step combines endocrine diagnosis, fracture history, bone quality assessment, biochemical profile, and fall-risk evaluation to support individualized fracture-risk stratification and personalized management.

Additional red flags include vertebral fractures despite osteopenic or normal bone mass, Z-score ≤ -2.0, inadequate response to anti-osteoporotic therapy, long-term glucocorticoid exposure, symptoms of thyrotoxicosis, features of Cushing syndrome, hypogonadal symptoms, nephrolithiasis or hypercalcemia, diabetes with complications, acromegalic features, and resistant hypertension suggestive of primary aldosteronism. These clinical features should prompt a structured evaluation aimed at identifying potentially reversible endocrine causes of skeletal fragility (**Figure 3**).

A pragmatic first-line biochemical panel should include serum calcium, albumin, phosphate, creatinine with estimated glomerular filtration rate, alkaline phosphatase, 25-hydroxyvitamin D, parathyroid hormone, thyroid-stimulating hormone, full blood count, liver function tests, HbA1c or fasting glucose, and sex hormone assessment when clinically appropriate. In men, morning testosterone with luteinizing hormone is usually required when hypogonadism is suspected. In premenopausal women, estradiol, follicle-stimulating hormone, luteinizing hormone, prolactin, and pregnancy-related considerations may be relevant. **Table 3** provides a practical laboratory framework. This initial biochemical assessment allows identification of common endocrine drivers and guides subsequent targeted investigations.

**Table 3:** First-line and targeted laboratory assessment.

Test	Purpose
Serum calcium and albumin	Hypercalcemia, corrected calcium, PHPT screening
Phosphate	Mineral metabolism disorders
Creatinine/eGFR	Renal contribution to bone disease and treatment selection
Alkaline phosphatase	Bone turnover, osteomalacia, liver disease differential
25-hydroxyvitamin D	Vitamin D deficiency/insufficiency
Parathyroid hormone	PHPT, secondary hyperparathyroidism, CKD-MBD evaluation
Thyroid-stimulating hormone	Hyperthyroidism or overtreatment with levothyroxine
HbA1c/fasting glucose	Diabetes-related skeletal risk
Morning testosterone, LH	Male hypogonadism
Estradiol, FSH, LH	Premature ovarian insufficiency, hypothalamic amenorrhea
24-hour urinary calcium	Hypercalciuria, PHPT, malabsorption, aldosteronism-related calcium loss
1 mg dexamethasone suppression test	Suspected endogenous glucocorticoid excess
IGF-1	Suspected acromegaly or GH-axis disorder
Aldosterone-renin ratio	Suspected primary aldosteronism
Serum protein electrophoresis/free light chains	Myeloma/MGUS differential when clinically indicated
Celiac serology	Malabsorption-related secondary osteoporosis
Serum phosphate	CKD-MBD, secondary hyperparathyroidism, mineral metabolism disorders

This table provides a practical overview of the recommended biochemical evaluation in patients with suspected endocrine-related secondary osteoporosis. It distinguishes between first-line laboratory tests, which should be routinely performed in the initial assessment, and targeted investigations guided by specific clinical or phenotypic suspicion. The listed tests aim to identify common endocrine and metabolic contributors to skeletal fragility, including disorders of calcium-phosphate metabolism, thyroid dysfunction, hypogonadism, glucocorticoid excess, diabetes mellitus, growth hormone abnormalities, and primary aldosteronism. This framework supports a systematic and clinically oriented diagnostic workflow, facilitating early detection of underlying conditions and appropriate risk stratification.

Targeted investigations should be phenotype-driven. A 1 mg overnight dexamethasone suppression test, late-night salivary cortisol, or urinary free cortisol may be used when Cushing syndrome is suspected. Twenty-four-hour urinary calcium is relevant in primary hyperparathyroidism, hypercalciuria, malabsorption, and suspected aldosteronism-related calcium wasting. Aldosterone-renin ratio should be considered in resistant hypertension, hypokalemia, adrenal incidentaloma, or hypertension with unexplained skeletal fragility. Insulin-like growth factor 1 should be measured when acromegaly is suspected. A phenotype-driven approach is essential to optimize diagnostic efficiency and avoid unnecessary testing.

Imaging should include DXA of lumbar spine and hip, with distal one-third radius in suspected primary hyperparathyroidism. When available, adjunctive imaging modalities such as vertebral fracture assessment, lateral spine morphometry, 3D-DXA reconstruction, and REMS may provide additional information regarding skeletal fragility and bone quality. These techniques may be particularly useful in patients with endocrine disorders characterized by a mismatch between BMD and fracture risk or when conventional densitometric findings appear discordant with the clinical phenotype. Vertebral fracture assessment or spine radiography should be considered in glucocorticoid exposure, acromegaly, height loss, back pain, previous fracture, or unexplained BMD-fracture discordance. TBS may be particularly useful in diabetes, glucocorticoid excess, primary aldosteronism, acromegaly, and other conditions in which microarchitecture is likely to be impaired. Overall, imaging should be interpreted within the clinical and endocrine context, particularly in cases of discordance between densitometric findings and fracture occurrence.

## 6 Targeted management

Management of endocrine-related secondary osteoporosis requires a dual strategy targeting both the underlying endocrine disorder and skeletal fragility.

Correction of the underlying endocrine disorder is mandatory. In glucocorticoid-induced osteoporosis, the lowest effective glucocorticoid dose should be used, and steroid-sparing strategies should be considered when feasible. In hyperthyroidism, restoration of euthyroidism is the primary skeletal intervention. In hypogonadism, hormone replacement may be appropriate when indicated and not contraindicated. In primary hyperparathyroidism, parathyroidectomy should be performed when guideline criteria are met. In diabetes, management should address glycemic control, complications, hypoglycemia avoidance, and fall risk. In acromegaly, biochemical control and correction of hypogonadism are essential. In primary aldosteronism, adrenalectomy or mineralocorticoid receptor antagonists may improve both cardiovascular and skeletal risk. Effective control of the endocrine disorder is a fundamental step in reducing ongoing skeletal damage.

General skeletal measures include adequate calcium intake, vitamin D repletion, protein sufficiency, smoking cessation, moderation of alcohol intake, progressive resistance training, balance training, fall-risk reduction, visual correction, medication review, and management of sarcopenia. These measures are essential components of fracture prevention and should be systematically implemented in all patients.

Pharmacological treatment should be individualized according to fracture risk, endocrine phenotype, renal function, prior therapy, and patient preference. Treatment selection should also consider the predominant pathophysiological mechanism, such as high turnover, low bone formation, or impaired bone material properties. Bisphosphonates remain appropriate first-line agents for many high-risk patients. Denosumab is effective but requires planned sequential therapy when discontinued to avoid rebound bone loss and vertebral fractures. Anabolic agents, including teriparatide, abaloparatide, and romosozumab where available and appropriate, should be considered in very-high-risk patients, particularly those with vertebral fractures, multiple fractures, glucocorticoid-induced low bone formation, or fractures during antiresorptive therapy. This approach is particularly relevant in conditions characterized by impaired bone formation or severe microarchitectural deterioration. In glucocorticoid-induced osteoporosis, current ACR and ECTS recommendations support early pharmacological treatment in patients at moderate, high, or very high fracture risk, with anabolic therapy favored in selected very-high-risk cases (12, 13). In men, recent ESCEO guidance supports risk-based assessment and sequential strategies in very-high-risk individuals (16). The most robust evidence for pharmacological treatment in endocrine-related secondary osteoporosis is available for glucocorticoid-induced osteoporosis. In a randomized controlled trial comparing teriparatide with alendronate in glucocorticoid-induced osteoporosis, teriparatide produced greater increases in lumbar spine and hip BMD and was associated with fewer new vertebral fractures than alendronate, supporting the use of anabolic therapy in patients with severe skeletal fragility or predominant suppression of bone formation (33). Similarly, randomized evidence comparing denosumab with risedronate showed greater increases in lumbar spine and hip BMD with denosumab, with a comparable safety profile, indicating that denosumab may represent an effective antiresorptive option when appropriately followed by sequential therapy after discontinuation (34). More recently, a Bayesian network meta-analysis confirmed that bisphosphonates, denosumab, and teriparatide are all effective in glucocorticoid-induced osteoporosis, while teriparatide and denosumab showed greater efficacy in improving lumbar spine and femoral neck BMD and reducing vertebral fracture risk (34).

For other endocrine disorders, direct fracture-endpoint trials are more limited, and treatment decisions should therefore integrate evidence from primary osteoporosis trials with disease-specific pathophysiology and clinical risk. In high-turnover states, such as hyperthyroidism or primary hyperparathyroidism before definitive biochemical control, correction of the endocrine disorder remains the first therapeutic target, but antiresorptive therapy may be considered when fracture risk is high, when fragility fractures are present, or when definitive endocrine treatment is delayed. In low-formation states, such as glucocorticoid excess or severe hypogonadism with vertebral fractures, anabolic or sequential anabolic–antiresorptive strategies may be biologically appropriate in very-high-risk patients. In diabetes mellitus, acromegaly, and primary aldosteronism, treatment should not rely exclusively on BMD changes, because fracture risk may be driven by impaired bone quality, vertebral fragility, fall risk, and disease-specific metabolic abnormalities. In CKD-MBD, antiresorptive and anabolic therapies require particular caution because bone turnover may be high, low, or mixed, and drug selection should be guided by renal function, mineral metabolism, PTH levels, and specialist evaluation (35).

Across endocrine disorders, treatment response should not be interpreted solely by changes in densitometric parameters; incident fractures, vertebral imaging, disease control, biochemical markers, adherence, fall risk, and TBS where available should be integrated. Overall, management should be dynamic and continuously re-evaluated based on fracture occurrence, disease control, and evolving risk profile.

## 7 Knowledge gaps and future directions

Despite recent advances, several critical evidence gaps remain. First, most anti-osteoporotic treatment trials have focused on postmenopausal osteoporosis rather than endocrine-specific secondary osteoporosis. As a result, evidence for fracture reduction in many endocrine phenotypes is indirect and often based on BMD changes. This highlights the need for fracture endpoint–driven studies specifically addressing endocrine-related skeletal fragility. Second, standardized disease-specific fracture-risk algorithms are lacking. FRAX underestimation in diabetes and glucocorticoid exposure illustrates the need for models incorporating disease duration, hormonal activity, medication dose, microvascular complications, biochemical markers, and bone-quality indices. Such models would enable a more accurate and individualized estimation of fracture probability. Third, TBS and advanced imaging require further validation in endocrine diseases. Although TBS is clinically accessible and promising, thresholds for intervention are not uniformly defined across endocrine phenotypes. High-resolution imaging offers mechanistic precision but remains largely confined to research settings. The lack of standardized thresholds limits their integration into routine clinical decision-making. Fourth, primary aldosteronism deserves greater attention in bone research. Its high prevalence among hypertensive patients, potential reversibility, association with parathyroid hormone and urinary calcium abnormalities, and apparent microarchitectural phenotype make it a relevant model of endocrine bone fragility. Further research in this field may provide important insights into potentially reversible mechanisms of skeletal impairment. Future studies should prioritize mechanism-based therapeutic strategies and evaluate whether early, targeted interventions can modify fracture outcomes independently of changes in bone mass.

## 8 Conclusions

Endocrine-related secondary osteoporosis is a clinically important and potentially reversible cause of skeletal fragility. Its recognition is essential because fracture probability may be underestimated when assessment is limited to DXA-derived measures. Glucocorticoid excess, thyroid disease, hypogonadism, primary and secondary hyperparathyroidism, chronic kidney disease–mineral and bone disorder, diabetes mellitus, growth hormone disorders, and primary aldosteronism impair skeletal strength through distinct but overlapping mechanisms involving remodeling imbalance, mineral metabolism disturbance, osteoblast and osteocyte dysfunction, collagen alteration, microarchitectural deterioration, and increased fall risk. A multidimensional clinical model should combine endocrine phenotype, fracture history, vertebral imaging, BMD, TBS where available, biochemical assessment, and disease-specific risk factors. Management should simultaneously target the underlying endocrine disorder and the mechanisms driving skeletal fragility. This perspective shifts the clinical focus from a purely densitometric view to a mechanism-based assessment of endocrine-related skeletal fragility, supporting a more precise and personalized strategy for fracture prevention.
